# Shear Band Evolution under Cyclic Loading and Fatigue Property in Metallic Glasses: A Brief Review

**DOI:** 10.3390/ma14133595

**Published:** 2021-06-28

**Authors:** Xiaodi Wang, Shaojie Wu, Ruitao Qu, Zhefeng Zhang

**Affiliations:** 1 Shi-changxu Innovation Center for Advanced Materials, Institute of Metal Research, Chinese Academy of Sciences, 72 Wenhua Road, Shenyang 110016, China; wangxiaodi@ustb.edu.cn (X.W.); sjwu15b@imr.ac.cn (S.W.); 2 National Center for Materials Service Safety, University of Science and Technology Beijing, Beijing 100083, China; 3 State Key Laboratory of Solidification Processing, School of Materials Science and Engineering, Northwestern Polytechnical University, 127 West Youyi Road, Xi’an 710072, China

**Keywords:** metallic glass, shear band, fatigue property, fatigue crack, free volume

## Abstract

The fatigue damage and fracture of metallic glasses (MGs) were reported to be dominated by shear band. While there exist several reviews about the fatigue behavior of MGs, an overview that mainly focuses on shear bands under cyclic loading is urgent, and is of great importance for the understanding of fatigue mechanisms and properties. In this review paper, based on the previous research results, the shear band evolution under cyclic loading including shear band formation, propagation and cracking, was summarized and elucidated. Furthermore, one strategy of enhancing the fatigue property through manipulating the microstructure to suppress the shear band formation was proposed. Additionally, the applications of the effect of annealing treatment and processing condition on fatigue behaviors were utilized to verify the strategy. Finally, several future directions of fatigue research in MG were presented.

## 1. Introduction

Metallic glass (MG) is a relatively new class of metallic material, which is fabricated by the fast-cooling during solidification. This alloy lacks the long-range order characteristic and the crystalline defects including grain boundaries and dislocations. The unique structural feature makes some mechanical properties of MG obviously superior than conventional crystalline alloys, such as strength, hardness, elastic limit, wear resistance, etc. [1,2,3]. It is reported that the compressive strength of a Co-based MG can reach as high as ~6.0 GPa, which may be the highest in the reported large-sized metallic materials [3]. MG is considered as a potential structural material owing to the advantages of its mechanical properties. As the preparation technology advances and the research further develops, it is believed that the key problem such as the small critical size and poor plasticity will be overcome, and then MG will be actually applied in the future.

It is estimated that ~90% of all mechanical failures in the structural materials are caused by fatigue [4]. Thus, the fatigue property is an important evaluation index before a new structural material application. Regrettably, the fatigue performance of bulk MG was firstly reported until 1998 [5], several decades later than the birth of MG material. One main reason is that in the early stage, the geometry of MG was restricted to thin ribbons, foils and films with thickness of less than millimeter scale, thus the specimen size cannot well meet the requirements of fatigue testing. In addition, much less attention was focused on the fatigue research of MG, compared with the large efforts in investigating the mechanical behaviors under monotonic loading. The relatively few fatigue studies make the comprehensive understanding of fatigue property and corresponding mechanisms lacking.

**Figure 1:**
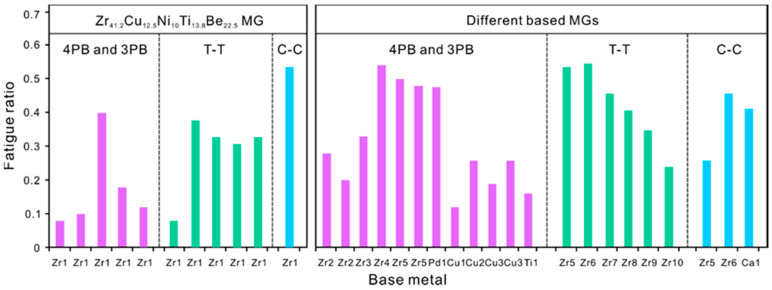
Statistical results of fatigue ratio in various metallic glasses (MGs) tested under different loading including four-point bending (4PB), three-point bending (3PB), tension–tension (T-T) and compression–compression (C-C) fatigue loading. The fatigue ratios are termed as the fatigue endurance limit divided by the fracture strength. The data for this figure were listed in Table 1.

The fatigue properties of MGs in terms of fatigue ratio were summarized here by the literature research [5,6,7,8,9,10,11,12,13,14,15,16,17,18,19,20,21,22,23,24,25,26,27,28,29,30]. The statistical results of fatigue ratio in various MGs tested under different loading modes were plotted in Figure 1 and Table 1. It is easily seen that the fatigue property of MG exhibits two typical characteristics: possibly low fatigue ratio and large fatigue scatter. A solid evidence for the first characteristic is that the fatigue limit of the earliest commercial Vitreloy-1 MG with the composition of Zr_41.2_Ti_13.8_Cu_12.5_Ni_10_Be_22.5_ was reported to be as low as ~150 MPa and its fatigue ratio based on the stress range is ~0.08, which is far lower than that of conventional crystalline metals [6]. However, using the same material and under different loading modes, the fatigue ratios of Vitreloy-1 MGs were soon found to range from ~0.08 to ~0.5 [5,8,9,10,11,15,16,17]; even using the same material and under identical loading mode, it is reported that the fatigue ratios of some MGs still show large difference, which may be mainly associated with the glassy microstructure [9,15].

Considering the intrinsic correlation between material property and mechanism, investigating the fatigue mechanism of MG will facilitate the understanding of fatigue property. Without the dislocations and grain boundaries, the inhomogeneous plastic deformation of MG occurs in the form of atomic clusters operation at room temperature, eventually leading to the generation of thin shear band. Since the large strain is highly localized in the shear band, the shear band is considered to be a favorable site for the nucleation and growth of fatigue crack. The shear-band traces have been obviously observed near the fatigue fracture surface in the extensive literature results [31,32], showing the important role of shear band in the fatigue damage. In addition, the good correspondence between the shear-band spacing on the sample surface and the fatigue-striation spacing on the fatigue fracture surface is well built [33]. Recent molecular dynamics (MD) simulations about the cyclic deformation of nano-scale MG further support the shear band mechanism, that is, the fatigue crack forms and propagates along the shear band [34]. So far, more attention is focused on the shear-band behavior under cyclic loading, while several important issues still exist:

(1) It is known that under monotonic loading, the shear band forms until the applied stress reaches the yield strength. Under cyclic loading, can the shear band form with the applied stress lower than the yield strength?
(2) How does the shear band propagate under cyclic loading?
(3) How does the shear band evolve into the fatigue crack? And what is the critical condition for the cracking?

Solving these questions will deepen the understanding of fatigue mechanism and fatigue property of MG, which is beneficial for the engineering application in the future. On the other hand, the study on the shear band behavior under cyclic loading will also enrich the shear-band theory of MG from the view of fundamental research.

In contrast to the previous reviews about the fatigue of MG [35,36,37], the topic of the current review article is dedicated to the progress of shear-band evolution under cyclic loading in MG and the improvement of fatigue property through controlling the shear banding behavior. The main frame was arranged in the following ordering. Firstly, the fatigue mechanism mediated by the shear band in MG was briefly discussed; then, the shear-band propagation and cracking behaviors were clarified, respectively; lastly, based on the shear banding behavior, a new strategy for enhancing the fatigue property was proposed and the validity of this strategy was further verified through two applications.

## 2. Shear Band-Mediated Fatigue Cracking Mechanism

The fatigue mechanism of MG was investigated based on the fatigue damage observations on the sample surface and fractography analyses. Similar to crystalline metals, the casting or processing defects usually exist in the fatigue crack initiation region of MG. Due to the large stress concentration near the defects, the fatigue crack preferentially forms from the defects. However, some studies reported no defects observed after fatigue fracture [7,21], implying another fatigue crack initiation mechanism. As the plastic deformation of MG is usually mediated by shear band at room temperature and shear band is weak owing to its high strain localization, the shear band is considered to be responsible for the fatigue cracking mechanism of MG, just like the slip band mechanism in crystalline metals. However, it is known that under monotonic loading the shear band can form only when the applied stress reaches the yield strength [38], this raises the query that whether the shear band can form under cyclic loading in the elastic regime.

**Figure 2:**
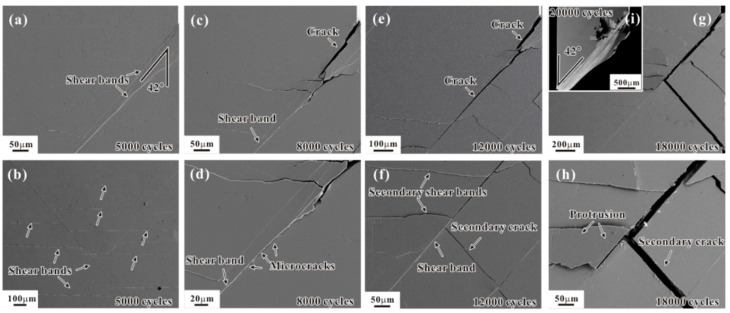
Fatigue damage morphologies under different cycles [42]: (**a**,**b**) N = 5000 cycles; (**c**,**d**) N = 8000 cycles; (**e**,**f**) N = 12,000 cycles; (**g**,**h**) N = 18,000 cycles; (**i**) N = 20,000 cycles.

Shear band traces on the sample surface as well as vein patterns due to the shear banding dominated fracture have been reported in different MGs after high-cycle fatigue [28,31,33]. This provides an indirect evidence for the shear band formation in the elastic cyclic loading regime. In addition, the experimental and simulation results suggest that the cyclic loading promotes the proliferation of free volume [39,40,41]. The increased free volume may induce the formation of shear band. However, no direct evidence of shear band formation under cyclic loading in the elastic regime was given. For clarifying this puzzle, Wang et al. [42] designed the quasi-in situ compression tests under cyclic loading with the maximum stress lower than the yield strength and captured the shear band evolution process by SEM observation. As shown in Figure 2a, the shear band emerged from the specimen edge before the fatigue crack formation, confirming the shear band formation under cyclic stress lower than the yield strength. Interestingly, much dense shear bands were seen in another side of this specimen, indicating the positive role of cyclic loading on the shear band initiation.

With increasing the cycles to 8000, the fatigue crack seems to initiate from the onset of shear band, as shown in Figure 2c. And some microcracks emerge inside the shear band ahead of fatigue crack tip. Probably, the microcracks would merge into the main fatigue crack, leading to the extension of fatigue crack. After the long cycles, the main fatigue crack becomes wider and longer, and some secondary cracks were also generated (Figure 2g,h). Eventually, the fatigue fracture occurs along the main shear band in a shear mode. Above results well demonstrate the shear band-mediated fatigue cracking mechanism of MG.

**Figure 3:**
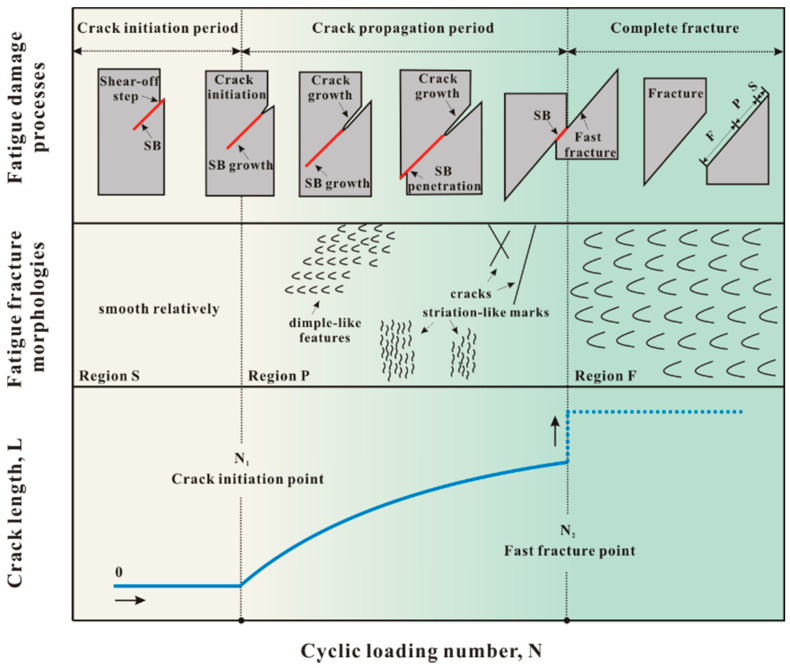
Comprehensive diagram of the shear band-mediated fatigue mechanism of MG [42].

The fatigue failure processes of material are usually divided into three stages, i.e., fatigue crack initiation, fatigue crack propagation and final fracture. The schematic diagram of fatigue failure process of MG was given based on these three stages, to further improve the understanding of shear band-mediated fatigue mechanism, as shown in Figure 3. With the fatigue deformation and damage proceeding, the shear band initiates and grows, then the fatigue crack initiates and grows along the shear band, and lastly the shear band fractures. The stages of shear band-mediated fatigue cracking on the sample surface correspond well with the regions on the fatigue fracture surface, i.e., the crack propagation region, the fast fracture region and the smooth region.

## 3. Shear Band Propagation under Cyclic Loading

### 3.1. Shear Band Propagation Mode under Cyclic Loading

**Figure 4:**
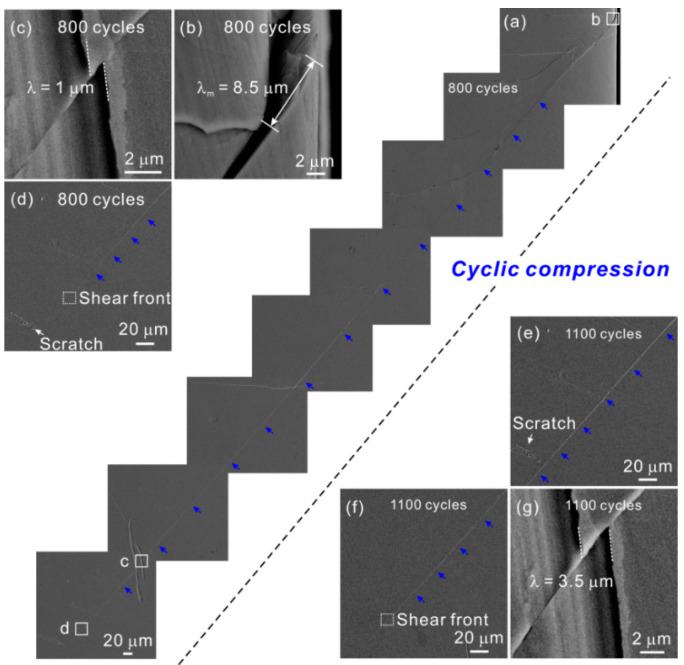
Shear band propagation after different cycles of cyclic compression [45]. (**a**) Overall apearance of the inserting shear band after 800 cycles. (**b**) Measurement of maximum shear offset after 800 cycles. (**c**,**g**) Measurement of the local shear offset at the same position of shear band after 800 and 1100 cycles, respectively. (**d**–**f**) Growth of the shear band tip from 800 cycles to 1100 cycles.

There are many studies about the propagation of shear band under monotonic loading [38,43,44]. Qu et al. [38] measured the shear offsets in different positions of shear band by using surface scratches as “rulers” and proved the existence of progressive propagation mode of shear band in monotonic compression. So far, the study about the shear band propagation under cyclic loading is rare, but of great importance. To determine the propagation mode of shear band, the evolution of an individual shear band under different loading cycles was observed with SEM and the shear offset was also measured by the scratch method [45], as shown in Figure 4.

After 800 cycles, one shear band stops inside the specimen (see Figure 4a,d). A straight scratch intersecting with the shear band is sheared off by the shear band and the value of local shear offset is ~1 μm. After 1100 cycles, this shear band continues to grow along the fixed shear direction, but still not transects the specimen, as evidenced by the position of shear front (see Figure 4f). The value of the local shear offset in the same position of the shear band increases to ~3.5 μm (see Figure 4g). It seems that the shear band progressively grows under cyclic loading.

**Figure 5:**
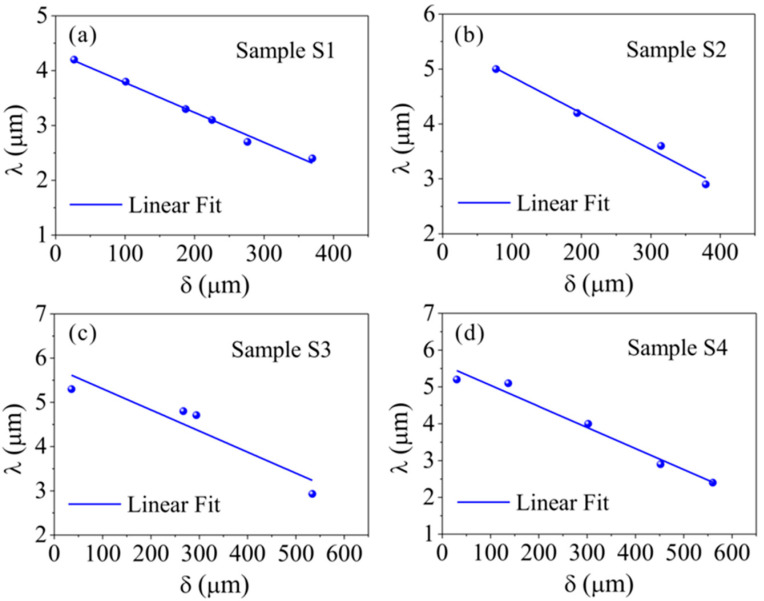
Local shear offset (*λ*) as a function of the distance from the origin site of shear band (*δ*) for shear bands from four samples (labeled as S1–S4) under cyclic compression [45]: (**a**) Sample S1; (**b**) Sample S2; (**c**) Sample S3; (**d**) Sample S4.

**Figure 6:**
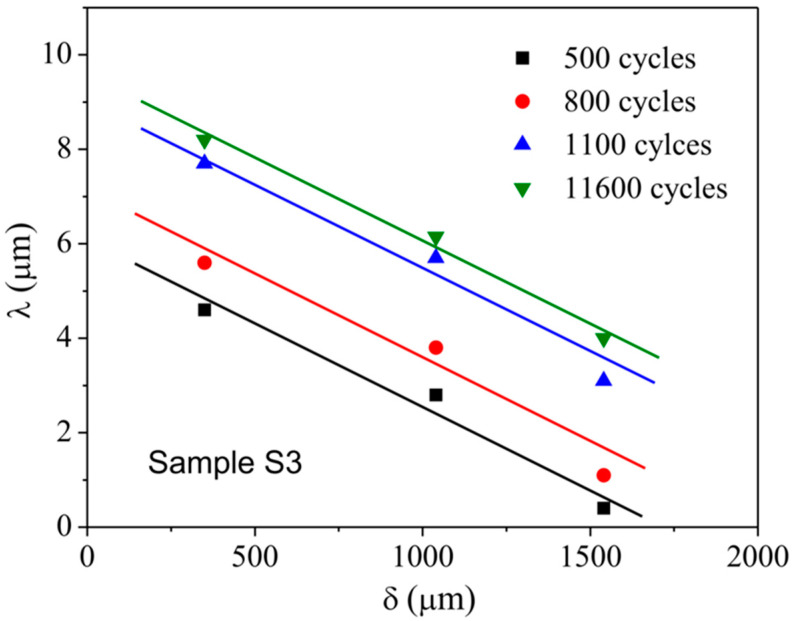
Local shear offset (*λ*) as a function of the distance from the origin site of shear band (*δ*) for one specific shear band after different loading cycles [45].

To verify the progressive mode of shear band under cyclic loading, the authors measured the relations between the local shear offset and the position along the shear band in different samples [45]. Obviously, the variation trends in different shear bands are similar, i.e., the local shear offset linearly decreases with increasing the distance from the origin site of shear band, as shown in Figure 5. Furthermore, the linear trend remains unchanged with increasing the loading cycles, as shown in Figure 6. The results give a direct evidence for the progressive mode of shear band propagation under cyclic loading.

### 3.2. Mechanism of Shear-Band Progressive Propagation under Cyclic Loading

During the progressive propagation of shear band under monotonic loading, the applied stress increases with increasing the strain, indicating the apparent “work-hardening” behavior [38]. In contrast, applying the identical cyclic stress level can also induce the progressive propagation of shear bands, which indicates that the “plastic softening” occurs under cyclic loading in MG. This phenomenon is explained by the effect of cyclic loading on microstructural change. Cameron et al. [39] performed the MD simulation of MG and found that the cyclic loading increased the free volume level. Using the same method, Li et al. [46] found that the cyclic loading induced the rejuvenation with the increase of free volume. The increased free volume under cyclic loading is one reason for the “cyclic plastic softening” behavior.

**Figure 7:**
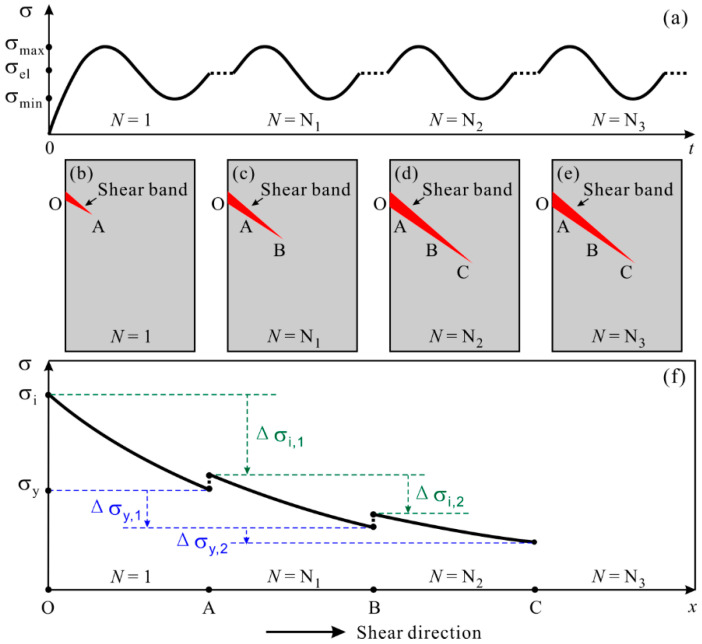
Schematic diagram illustrating the mechanism of progressive shear band propagation under cyclic compression of MGs [45]. (**a**) Illustration of applied cyclic stress (σ) as a function of loading time (t). (**b**–**e**) Illustrations of shear band propagation with increasing the number of loading cycle. (**f**) Possible stress distribution along the shear direction of Ox. *σ*_i_ and *σ*_y_ are the critical stresses for shear band initiation and propagation.

The mechanism of shear-band progressive propagation under cyclic loading can be illustrated in Figure 7. There exists the stress concentration at the edge of specimen (point O). When the stress at point O reaches the critical stress for shear band initiation, *σ*_i_, the shear band initiates. The shear band can propagate in the path with the stress exceeding the critical stress for shear band propagation, *σ*_y_ [47]. With the distance away from the point O, the stress gradually decreases. The shear band stops propagating at point A where the stress is equal to *σ*_y_. Despite of the constant cyclic stress level, the increasing loading cycles promote the generation and accumulation of free volume, probably ahead of shear band tip. The *σ*_i_ and *σ*_y_ are expected to be reduced and the stress at point A can reach *σ*_i_ again. Then the shear band propagates to point B, just like the propagating process from O to A. The following process of shear band propagation is repeated, leading to the mode of shear-band progressive propagation under cyclic compression.

## 4. Shear Band Cracking under Cyclic Loading

### 4.1. Shear Band Cracking Observation under Cyclic Loading

**Figure 8:**
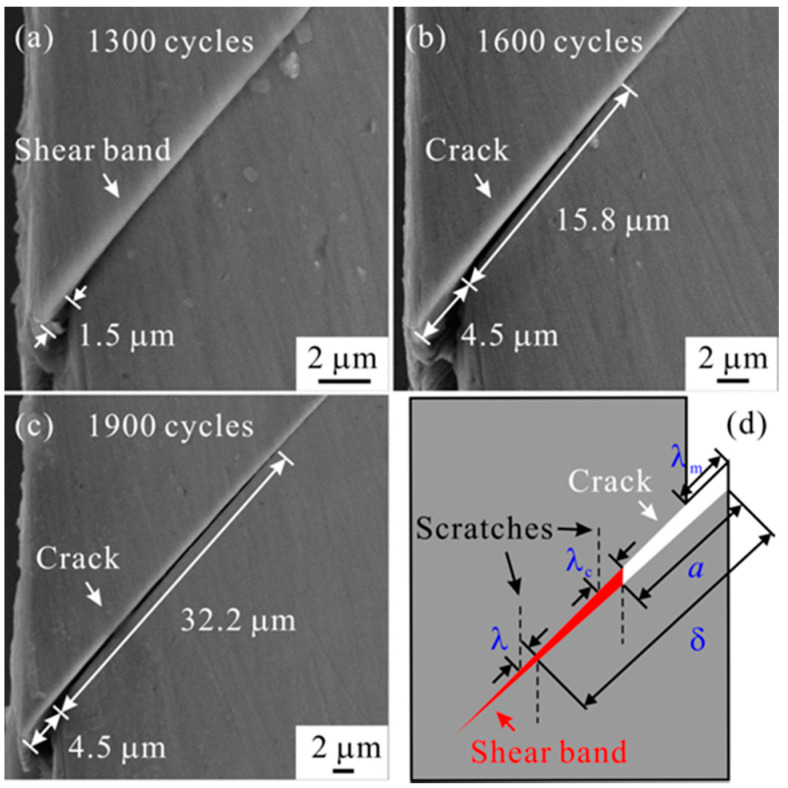
SEM observations on the damage evolution under different cycles of (**a**) 1300, (**b**) 1600 and (**c**) 1900. (**d**) Illustration of the shear band and shear-band crack and the definitions of some parameters, i.e., the local shear offset (*λ*), the distance (*δ*) from the origin site of shear band, the maximum shear offset (*λ*_m_), the critical shear offset for cracking (λ_c_), and the crack length (a) [48].

Because the high plastic strain is localized into the shear band, the fatigue crack is prone to originate from the shear band. Figure 8 displays the damage evolution process under cyclic loading [48]. The shear band initiates, propagates and cracks after different cycles. This process is consistent with the results in Ref. [42]. Here, to quantitatively study the shear-band cracking behavior, several key parameters were defined. For example, the local shear offset at the crack tip is termed as the critical shear offset for shear-band cracking, *λ*_c_; the shear offset at the onset of shear band is termed as maximum shear offset, *λ*_m_.

### 4.2. Critical Condition for Crack Initiation and Propagation under Cyclic Loading

**Figure 9:**
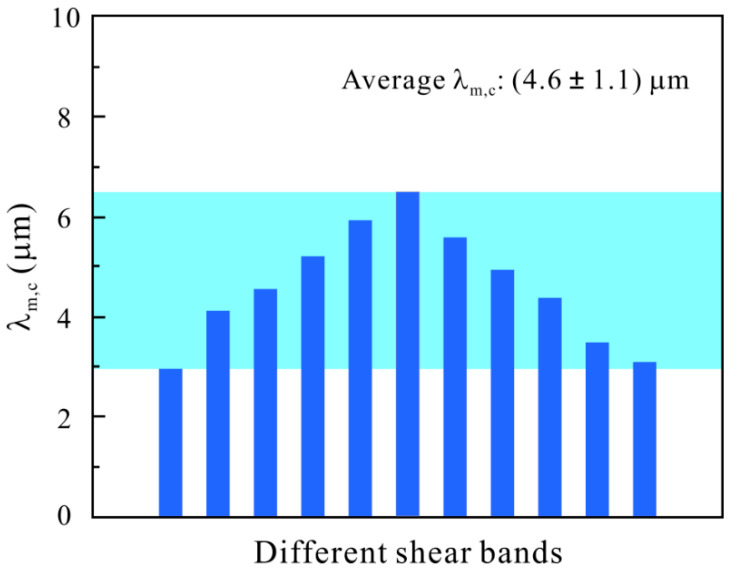
Critical shear offset for crack initiation, *λ*_m,c_, in different shear bands after cyclic compression [48].

The concept of “critical shear offset” was usually utilized as the critical condition for shear fracture under monotonic loading [49,50]. The researchers measured the smooth region on the fracture surface or the height of shear step along the shear band to obtain the critical shear offset. For the cyclic loading, the shear-band cracking occurs firstly at the onset of shear band (see Figure 8b), so the maximum shear step at the onset of shear band upon the fatigue crack initiation was measured as the critical shear offset for fatigue crack initiation, *λ*_m,c_. The critical condition for fatigue crack initiation in term of shear offset is ~(4.6 ± 1.1) μm, as shown in Figure 9.

**Figure 10:**
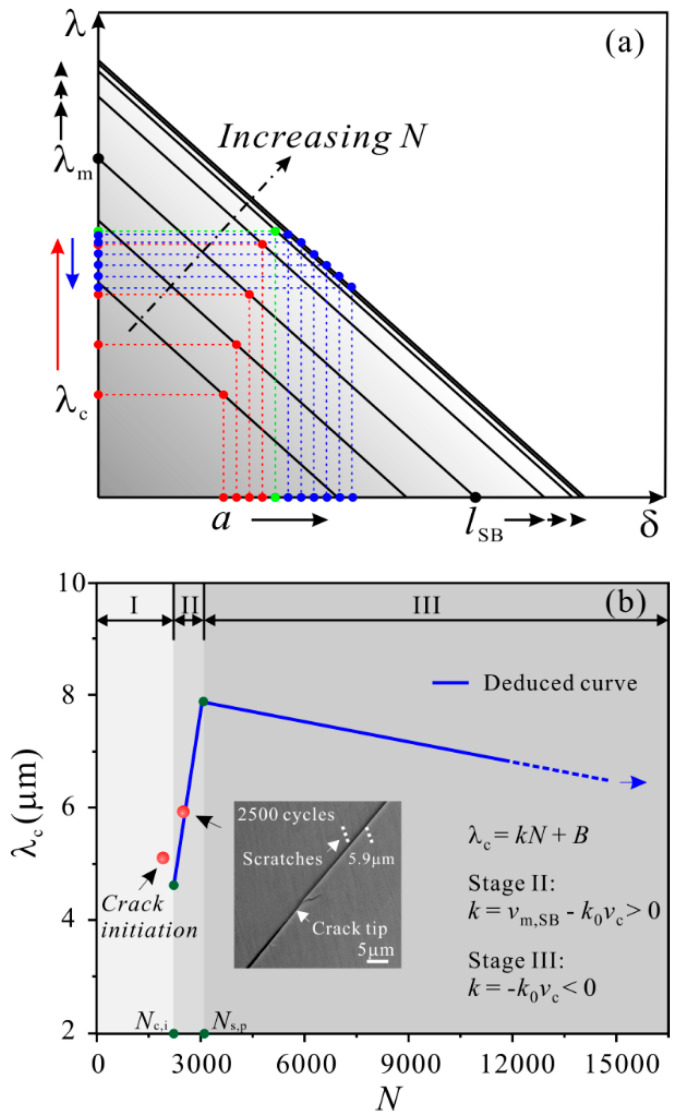
(**a**) Schematic illustrations of *λ* as a function of *δ* under different cycles, showing the detailed derivation process of *λ*_c_; (**b**) plot of *λ*_c_ as a function of *N* [48].

On the other hand, the critical shear offset for shear-band cracking, *λ*_c_ during fatigue crack propagation was obtained. The derivation process of *λ*_c_ was illustrated in Figure 10a and the detailed equation derivation can be viewed in Ref. [48]. The *λ*_c_ as a function of loading cycles was shown in Figure 10b. The *λ*_c_ firstly increases owing to the higher rate of shear band propagation than that of fatigue crack propagation, but then decreases because the propagation of shear band ceases. This indicates that the cyclic loading firstly suppresses but eventually promotes the shear-band cracking. The variation trend of *λ*_c_ was explained based on the competition between the accumulation of dilatation damage within the shear band and the release of stress concentration due to plastic shearing.

## 5. Strategy for Optimizing the Fatigue Property of MG

**Figure 11:**
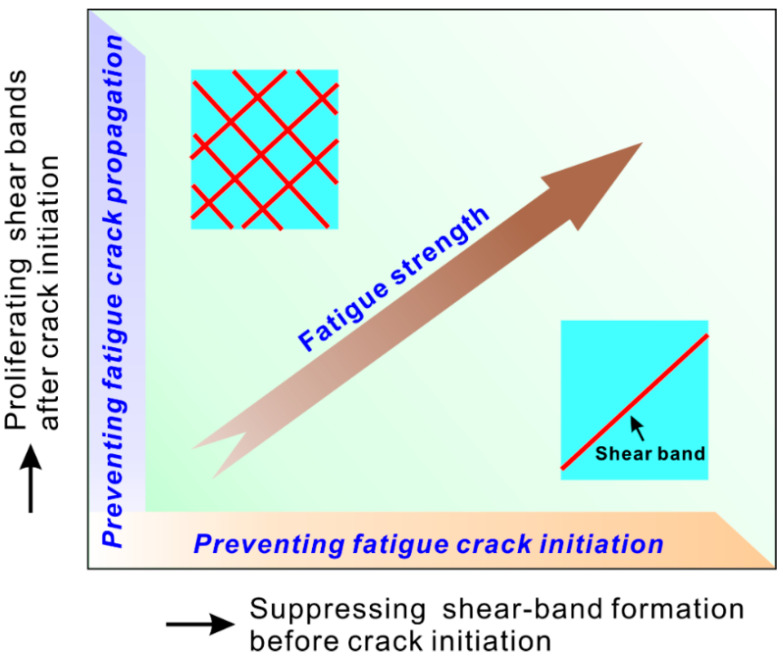
Two approaches to improve the fatigue strength of MGs: (1) preventing fatigue crack initiation via suppressing shear-band formation; (2) preventing fatigue crack propagation via promoting shear-band proliferation during fatigue crack propagation [53].

Based on the above results and discussion, controlling the shear banding behavior should be an effective way to enhance the fatigue property of MG. Indeed, in this view, several extrinsic methods such as coating, shot peening etc. have been applied to enhance the fatigue property [51,52]. More importantly, two intrinsic methods of enhancing the fatigue property of MG were summarized in Figure 11. One is to proliferate the shear bands after fatigue crack initiation and then to prevent the fatigue crack propagation. This method has been verified in high-toughness MGs [6,7]. In contrast to this method, Wang et al. [53] proposed a new strategy by properly tailoring the microstructure to increase the resistance to shear band formation and then preventing the fatigue crack initiation from the shear band. This new strategy can be verified by two applications.

### 5.1. Application I: Annealing Treatment

One key to achieve the new strategy is to increase the resistance to shear band formation. The annealing treatment can relax the microstructure and suppress the formation of the shear band [54,55]. The annealing is also controllable, nondestructive and easy to conduct in practice. However, it should be noted that over annealing may cause partial crystallization. The crystalline phases probably prevent the shear band propagation and promote the proliferation of shear bands [19]. The increased shear bands will provide more chances for the fatigue crack initiation, deteriorating the fatigue property. Thus, the annealing parameters should be preliminarily selected by examining the relaxation and crystallization curves of MG, so that the sample can be fully relaxed and no crystallization will occur during annealing [53]. Then the X-ray diffraction test should be conducted to further confirm the amorphous structure.

Another key to achieve the new strategy is to obtain the optimum microstructure corresponding to the enhanced fatigue property. Due to the lack of long-range order structure, the microstructure of MG is difficult to be directly characterized. As the mechanical property is closely related to the microstructure, the mechanical property can be used to inversely estimate the microstructure.

**Figure 12:**
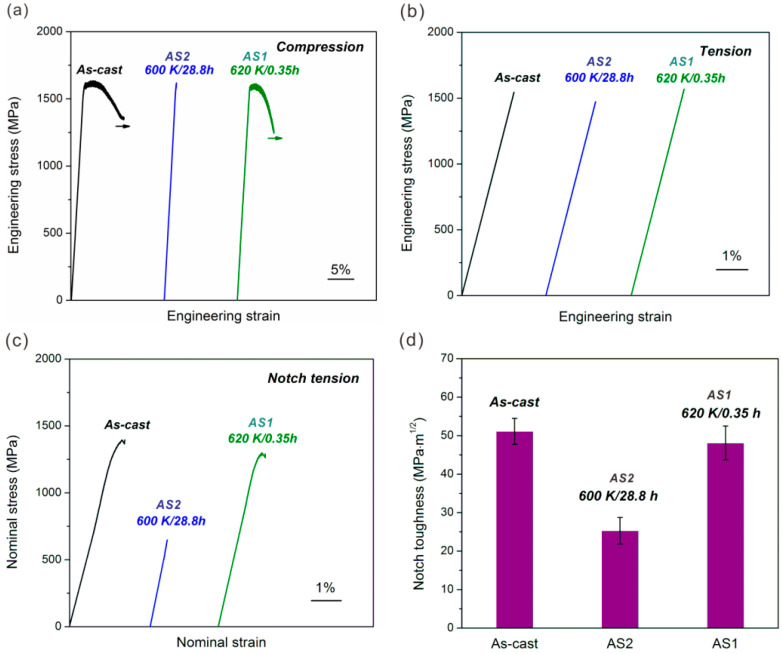
(**a**,**b**) Typical compressive and tensile engineering stress–strain curves of the as-cast and annealed MG samples; (**c**,**d**) notch tensile nominal stress–strain curve and notch toughness of the as-cast and annealed MG samples [53].

The simple tension, compression and notch tension properties could serve as the feedbacks to evaluate the casting and annealing MGs with different microstructures. The elastic limit measured from compression represents the resistance for shear band formation, the plasticity measured from compression is related to shear-band density and the notch toughness measured from notch tension can characterize the tolerance for fatigue failure. The better microstructure after annealing should correspond to the increased elastic limit, reduced compressive plasticity, and slightly decreased notch toughness. Figure 12 is the results of tension, compression and notch tension of casting and annealing MGs. The elastic limit and tensile strength were enhanced while the platform-plasticity was reduced and the toughness was slightly reduced after AS1 annealing treatment. This AS1 annealed state is expected be the structural state for the enhanced fatigue property of MG.

**Figure 13:**
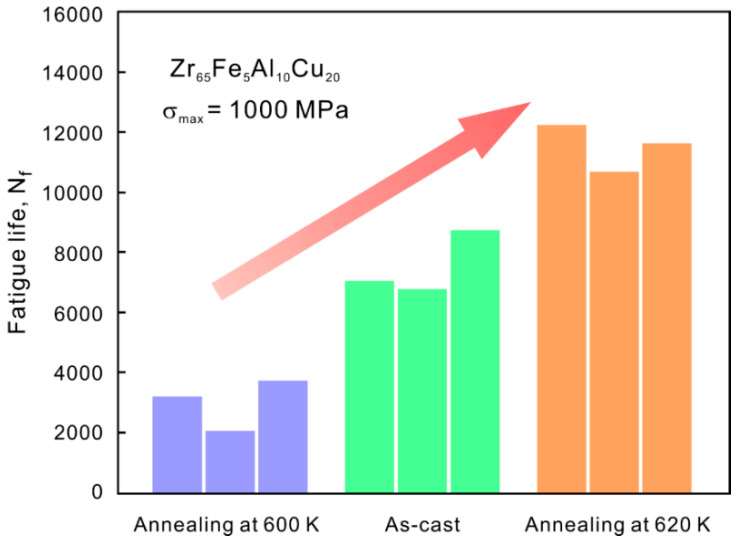
Fatigue life of the as-cast and annealed samples under *σ*_max_ = 1000 MPa [53].

**Figure 14:**
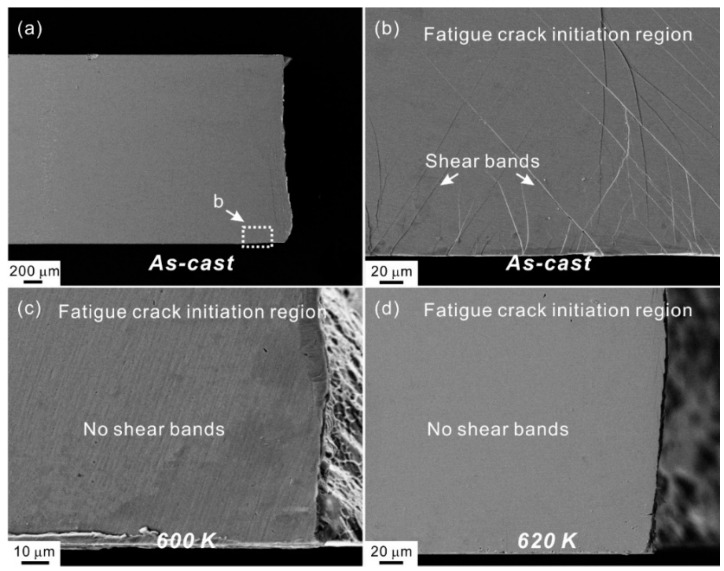
SEM observations on the tensile-side surface near the fatigue crack initiation regions of (**a,b**) as-cast, (**c**) 600 K (AS2) and (**d**) 620 K (AS1) annealed samples after fatigue fracture, respectively [53].

The authors conducted the fatigue tests under one identical stress level to compare the fatigue life of the casting and annealing samples [53]. The test results were presented in Figure 13. It is indeed seen that the fatigue life of AS1 annealed samples is ~50% higher than that of the as-cast one. In order to further confirm whether the enhancement of fatigue life is related to the shear-band suppression, the damage features in the fatigue crack initiation regions of the casting and annealing samples were examined. A large number of shear bands emerged from the edge of the casting sample, while nearly no shear bands were seen after AS1 annealing, as shown in Figure 14, demonstrating the suppression of shear band. In summary, annealing treatment has been employed as an application to verify the proposed strategy, which can relax the glassy structure and increase the resistance for shear band formation, and finally improve the fatigue property of MG.

### 5.2. Application II: Processing Condition

The processing conditions such as the casting temperature and cooling rate can largely affect the microstructure of MG, eventually inducing different shear-band behaviors and mechanical properties [56,57]. Adjusting the processing conditions was selected as another effective method to support the proposed strategy [58]. Two batches of MGs with different processing conditions (termed as Vit-105A MG and Vit-105B MG) were selected to investigate the uniaxial and cyclic compression properties. Thermal analysis suggests that compared with Vit-105B MG, the Vit-105A MG is in the lower free volume level and more relaxed state.

**Figure 15:**
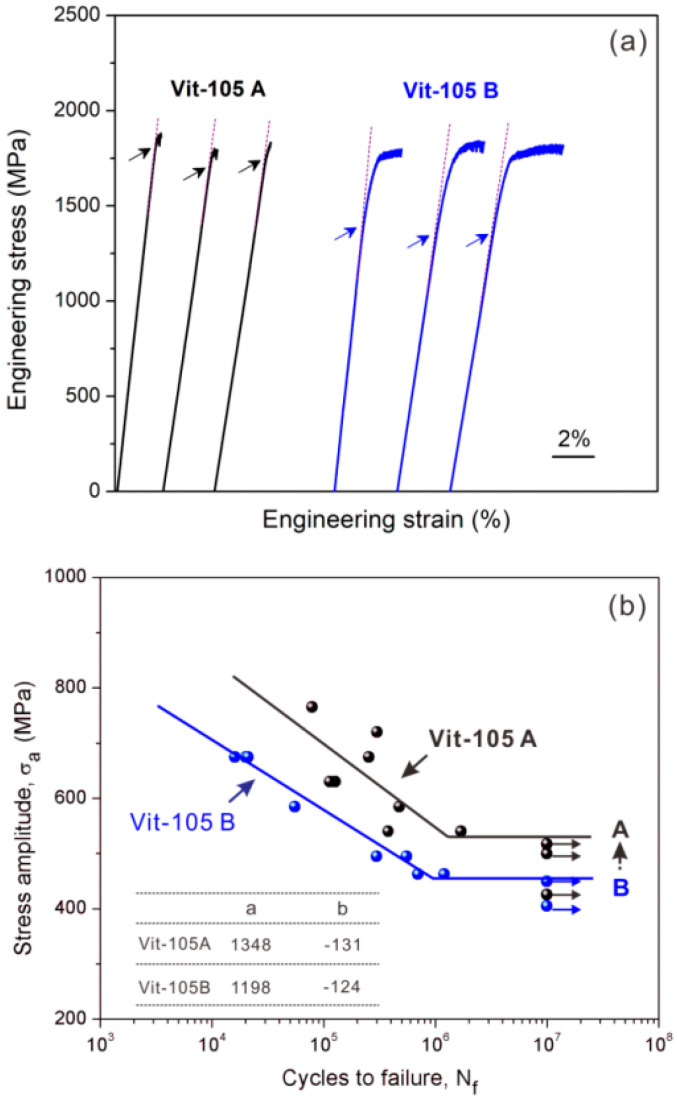
(**a**) Compressive engineering stress-strain curves of different batches of metallic glasses (MGs), i.e., Vit-105 A and Vit-105 B; (**b**) stress-life (*S-N*) fatigue data for the Vit-105A and Vit-105B MGs [58].

Figure 15 is the results of uniaxial compression and cyclic compression. Obvious, these two MG show different elastic limit (as indicated by the arrows) and plasticity, that is, the elastic limit of Vit-105A MG is higher and its plasticity is lower. It is noted that the plasticity of Vit-105A MG is limited, but is not reduced to zero, which can ensure the shear band mechanism.

**Figure 16:**
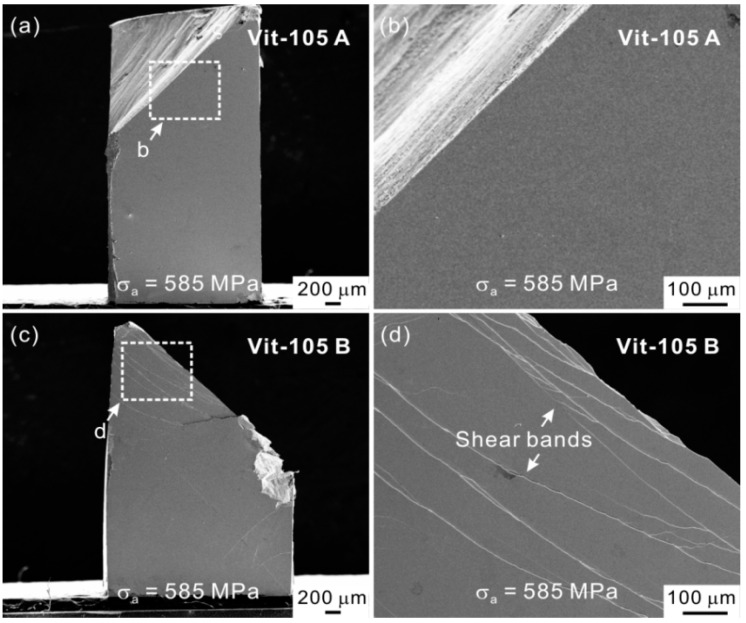
(**a**,**c**) Macroscopic damage morphologies of Vit-105A and Vit-105B MGs under an identical stress amplitude; (**b**,**d**) magnified images of the rectangle regions in (**a**,**c**), showing different shear-band features [58].

Based on the proposed strategy and the annealing treatment result above, the Vit-105A MG with higher elastic limit and reduced plasticity should possess the better fatigue property due to the higher resistance to shear band formation. The stress-life (*S-N*) fatigue data indeed proves that the fatigue endurance limit and fatigue life of Vit-105A MG are higher than the other MG (Figure 15b). Furthermore, the comparison of fatigue damage features after fatigue fracture in Vit-105A and Vit-105B MGs indicates that the shear band formation under cyclic loading is suppressed in Vit-105A MG (see Figure 16). Hence, the results about the processing conditions are consistent with the new strategy of enhancing the fatigue property through tailoring the microstructure to increase the resistance to shear band formation.

## 6. Conclusions

The shear band plays a key role in the fatigue damage behavior in MGs. In this review paper, the progress of the shear band evolution under cyclic loading and fatigue property were summarized, mainly focusing on our recent results. The conclusions are as follows:

The quasi-in situ experimental results directly confirmed that the shear band can form under cyclic loading lower than the yield strength. The fatigue crack was found to initiate and propagate along the shear band, demonstrating the shear band-mediated fatigue cracking mechanism. The shear band propagates in a progressive mode under cyclic loading, which indicates that the “cyclic plastic softening” occurs, in contrast to the apparent “work-hardening” behavior caused by the progressive propagation of shear band under monotonic loading. The critical conditions for fatigue crack initiation and propagation are obtained. The small critical shear offset for fatigue crack initiation may be one reason for the low fatigue property in MG.

A strategy for enhancing the fatigue property of MG through properly tailoring the microstructure to suppress the shear band formation and then preventing the fatigue crack initiation was proposed. This strategy was verified by two applications of annealing treatment and processing condition. The optimum microstructure with better fatigue property can be determined by simple mechanical tests including compression, tension and notch tension. The MG with relatively high elastic limit and a small amount of plasticity is expected to show the better fatigue property.

## 7. Unsolved Issues and Outlook

Although great efforts about the shear band evolution under cyclic loading and fatigue property have been made in recent studies, several important issues are still needed to solve in the future.

(1) There exists a controversy about the effect of cyclic loading on microstructure of MG [39,40,41,59,60,61,62,63,64]. That is, the cyclic loading may induce the structural relaxation or rejuvenation, and nanocrystallization. Even several simulation results indicate that there is no structural change after cyclic loading. So, more studies should be carried out to further identify the effect of cyclic loading on microstructure, using the advanced experimental techniques and simulation. Understanding this issue is beneficial for revealing the physical nature of the shear band initiation and propagation under cyclic loading.
(2) The proposed strategy of enhancing the fatigue property of MG is relatively phenomenological. The quantitative relationship among the initial microstructure, shear band, fatigue crack and fatigue property should be built, which will promote understanding of the shear band-mediated fatigue mechanism and provide the further theoretical basis for this proposed strategy.
(3) The large difference among the fatigue property of MG is caused by many external factors including materials quality, loading mode, stress ratio, specimen geometry, residual stress and so on [36,37,65]. Investigating the correlation between these factors and shear banding behaviors is a viable approach to further clarify the fatigue mechanism and property.

## Figures and Tables

**Table 1:** Summary of fatigue limits and ratios under varying fatigue loading modes in different MGs based on the literature reports [5,6,7,8,9,10,11,12,13,14,15,16,17,18,19,20,21,22,23,24,25,26,27,28,29,30].

Material	Label	Geometry (mm)	Test Configuration	Fatigue Limit (MPa)	Fatigue Ratio
Zr_41.2_Cu_12.5_Ni_10_Ti_13.8_Be_22.5_ [5]	Zr1	3 × 3 × 50 (S_1_ = 10; S_2_ = 20)	4PB	152	0.08
Zr_41.2_Cu_12.5_Ni_10_Ti_13.8_Be_22.5_ [8]	Zr1	3 × 3 × 40 (S_1_ = 10; S_2_ = 30)	4PB	190	0.10
Zr_41.2_Cu_12.5_Ni_10_Ti_13.8_Be_22.5_ [9]	Zr1	2 × 2 × 60 (S_1_ = 0; S_2_ = 48)	3PB	768	0.40
Zr_41.2_Cu_12.5_Ni_10_Ti_13.8_Be_22.5_ [9]	Zr1	2 × 2 × 60 (S_1_ = 0; S_2_ = 48)	3PB	359	0.18
Zr_41.2_Ti_13.8_Cu_12.5_Ni_10_Be_22.5_ [10]	Zr1	S_1_ = 0; S_2_ = 20	3PB	225	0.117
Zr_41.2_Cu_12.5_Ni_10_Ti_13.8_Be_22.5_ [11]	Zr1	∅4 × 20	T-T (smooth)	152	0.08
Zr_41.2_Cu_12.5_Ni_10_Ti_13.8_Be_22.5_ [15]	Zr1	∅2.98	T-T (Sharp notch)	703	0.38
Zr_41.2_Cu_12.5_Ni_10_Ti_13.8_Be_22.5_ [15]	Zr1	∅2.98	T-T (Sharp notch)	615	0.33
Zr_41.2_Cu_12.5_Ni_10_Ti_13.8_Be_22.5_ [16]	Zr1	∅2.98	T-T (Tapered notch)	567	0.31
Zr_41.2_Ti_13.8_Cu_12.5_Ni_10_Be_22.5_ [10]	Zr1	∅4	T-T (Tapered notch)	630	0.328
Zr_41.2_Ti_13.8_Cu_12.5_Ni_10_Be_22.5_ [17]	Zr1	-	C-C	1050	0.54
Zr_44_Cu_10_Ni_10_Ti_11_Be_25_ [18]	Zr2	2.3 × 2 × 85 (S_1_ = 30; S_2_ = 60)	4PB	550	0.28
Zr_44_Cu_10_Ni_10_Ti_11_Be_25_ [18]	Zr2	2.3 × 2 × 85 (S_1_ = 30; S_2_ = 60)	4PB	390	0.20
(Zr_58_Ni_13.6_Cu_18_Al_10.4_)_99_Nb_1_ [19]	Zr3	2 × 2 × 25 (S_1_ = 10; S_2_ = 20)	4PB	559	0.33
Zr_61_Ti_2_Cu_25_Al_12_ [7]	Zr4	3 × 3 × 25 (S_1_ = 10; S_2_ = 20)	4PB	880	0.54
Zr_52.5_Al_10_Ti_5_Cu_17.9_Ni_14.6_ [20]	Zr5	3.5 × 3.5 × 30 (S_1_ = 5; S_2_ = 20)	4PB	850	0.5
Zr_52.5_Al_10_Ti_5_Cu_17.9_Ni_14.6_ [21]	Zr5	2 × 2 × 25 (S_1_ = 10; S_2_ = 20)	4PB	816	0.48
Pd_79_Ag_3.5_P_6_Si_9.5_Ge_2_ [6]	Pd1	S_1_>4; S_2_>8	4PB	720	0.48
Cu_47.5_Zr_47.5_Al_5_ [12]	Cu1	3 × 3 × 25 (S_1_ = 10; S_2_ = 20)	4PB	224	0.12
(Cu_60_Zr_30_Ti_10_)_99_Sn_1_ [13]	Cu3	2.85 × 2.85 × 25 (S_1_ = 10; S_2_ = 20)	4PB	350	0.19
Cu_45_Zr_45_Ag_7_Al_3_ [22]	Cu2	3 × 3 × 25 (S_1_ = 5; S_2_ = 20)	4PB	386	0.26
(Cu_60_Zr_30_Ti_10_)_99_Sn_1_ [13]	Cu3	2.85 × 2.85 × 25 (S_1_ = 0; S_2_ = 20)	3PB	475	0.26
Ti_32.8_Zr_30.2_Ni_5.3_Cu_9_Be_22.7_ [14]	Ti1	2 × 7.2 × 30	3PB (Sharp notch)	290	0.16
Zr_76.6_Al_3.5_Cu_12.3_Ni_7.6_ [23]	Zr10	2.5 × 4 × 16	T-T (smooth)	300	0.24
Zr_52.5_Al_10_Ti_5_Cu_17.9_Ni_14.6_ [24]	Zr5	∅2.98	T-T (Sharp notch)	907	0.54
Zr_50_Al_10_Cu_40_ [25]	Zr8	∅2.98	T-T (Sharp notch)	752	0.41
Zr_50_Al_10_Cu_30_Ni_10_ [25]	Zr7	∅2.98	T-T (Sharp notch)	865	0.46
Zr_50_Cu_37_Al_10_Pd_3_ [26]	Zr6	∅2.98	T-T (Sharp notch)	983	0.55
Zr_55_Cu_30_Al_10_Ni_5_ [27]	Zr9	1 × 2 × 5	T-T (Sharp notch)	620	0.35
Zr_50_Cu_37_Al_10_Pd_3_ [28]	Zr6	∅5 × 10	C-C	874	0.46
Ca_65_Mg_15_Zn_20_ [29]	Ca1	4 × 4 × 4	C-C	126	0.42
Zr_52.5_Al_10_Ti_5_Cu_17.9_Ni_14.6_ [30]	Zr5	2 × 2 × 4	C-C	450	0.26

Note here S_1_ and S_2_ represent the inner and outer span during the bending fatigue test, respectively. The fatigue limit is summarized based on the stress range.
